# Supplementary material for: Dietary Fiber in Bilberry Ameliorates Pre-Obesity Events in Rats by Regulating Lipid Depot, Cecal Short-Chain Fatty Acid Formation and Microbiota Composition
Source: Nutrients. 2019 Jun 15;11(6):1350. doi: 10.3390/nu11061350 (PMC6627426; doi:10.3390/nu11061350)
Supplement: Supplementary file 1 [file nutrients-11-01350-s001.pdf]

Table S1. Primers used for Real-Time qPCR

mRNA	Forward primer	Reverse primer
Acc	*GGACAACACCTGTGTGGTAGAA*	*CGTGGGGATGTTCCCTCT*
Cpt1α	*ACAATGGGACATTCCAGGAG*	*AAAGACTGGCGCTGCTCA*
Fabp5	*TGCACCTTGGGAGAGAAGTT*	*AAGGTGCAGACCGTCTCAGT*
Fasn	*GGCATCATTGGGCACTCCTT*	*GCTGCAAGCACAGCCTTCTCT*
Mttp	*GCGAGTCTAAAACCCGAGTG*	*CACTGTGATGTCGCTGGTTATT*
Pparα	*TGCGGACTACCAGTACTTAGGG*	*GCTGGAGAGAGGGTGTCTGT*
Pparγ	*TCCTCCTGTTGACCCAGAGCAT*	*AGCTGATTCCGAAGTTGGTGG*
Scd-1	*GAAGCGAGCAACCGACAG*	*GGTGGTCGTGTAGGAACTGG*
TfIIb	*CCTGGCAGGAGTCCTATCTCT*	*ACCAGCAATATCCCCGATTT*
Ucp-1	*GCCTGCCTAGCAGACATCAT*	*TGGCCTTCACCTTGGATCT*

Table S2. Cecal pools of SCFAs, lactic and succinic acid (μmol) and serum concentration (μmol/L) of SCFAs in rats fed experimental diets (means ± SEM, n = 6).

Cecum (μmol)	LFD	HFD	LFD+BB	HFD+BB	Bilberry	HF	BB&HF
Total	138±28^{bc}	78±7^{c}	280±48^{a}	190±18^{ab}	<0.001	0.006	0.620
Acetic acid	90±17^{bc}	51±4^{c}	214±38^{a}	135±12^{ab}	<0.001	0.008	0.340
Propionic acid	21±5^{a}	7±1^{b}	25±3^{a}	16±2^{ab}	0.021	<0.001	0.390
Butyric acid	13±3^{ab}	7±0^{b}	18±3^{a}	19±3^{a}	0.001	0.320	0.190
Caproic acid	0.2±0^{ab}	0.1±0^{b}	0.2±0^{a}	0.2±0^{a}	0.001	0.440	0.480
Succinic acid	3±1^{b}	7±1^{ab}	11±3^{a}	12±1^{a}	<0.001	0.054	0.110
Valeric acid	3.2±0^{a}	0.9±0^{b}	2.6±0^{ab}	1.2±0^{b}	0.690	<0.001	0.300
Iso-butyric acid	2±0^{a}	1±0^{a}	1±0^{a}	1±0^{a}	0.150	0.063	0.150
Iso-valeric acid	3±1^{a}	2±0^{a}	1±0^{a}	1±0^{a}	0.087	0.240	0.240
Heptanoic acid	0.1±0^{a}	0.1±0^{a}	0.2±0^{a}	0.1±0^{a}	0.077	0.150	0.290
Lactic acid	3±1^{a}	3±1^{a}	6±1^{a}	4±1^{a}	0.190	0.690	0.270

Serum (μmol/L)	LFD	HFD	LFD+BB	HFD+BB	Bilberry	Fat	BB&HF
Total	705±201	808±126	1150±264	990±214	0.2	0.9	0.6
Acetic acid	552±155	701±107	874±171	816±162	0.2	0.5	0.5
Propionic acid	107±37	64±17	218±86	124±50	0.1	0.3	0.7
Iso-butyric acid	9±1	9±1	11±3	8±2	0.8	0.6	0.7
Butyric acid	19±5	19±4	27±6	29±6	0.07	0.4	0.9
Iso-valeric acid	15±3	13±2	13±2	10±3	0.4	0.5	0.9
Valeric acid	6±2	5±2	8±3	4±2	0.9	0.2	0.6

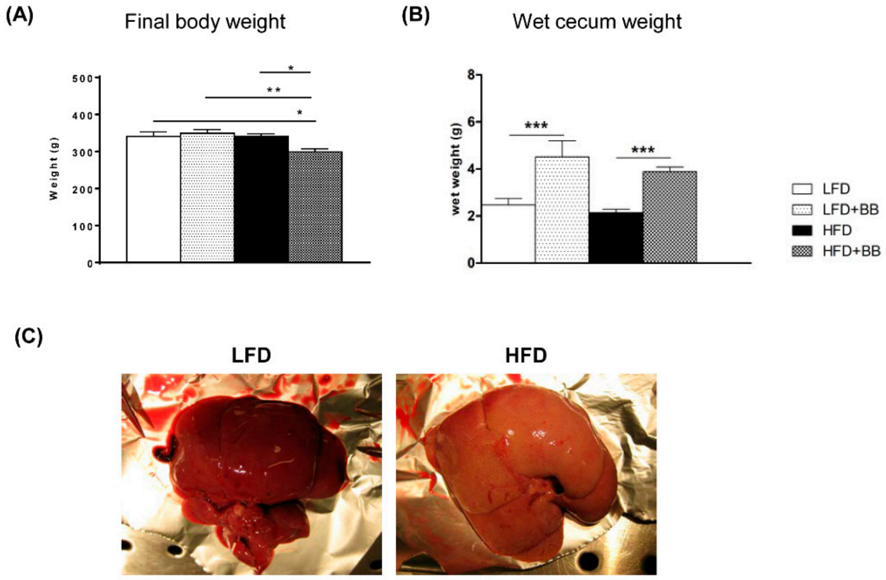

Figure S1. Final body weight, cecum weight and liver morphology.

LFD	
Bacteroides	0.16
Akkermansia	0.05
Clostridium	0.05
Parabacteroides	0.04
Ruminococcaceae Incertae Sedis	0.04
Parasutterella	0.03
Lachnospiraceae Incertae Sedis	0.02
Peptostreptococcaceae Incertae Sedis	0.02
Anaerotruncus	0.01
Family XIII Incertae Sedis	0.01
Lactobacillus	0.01
Oscillibacter	0.01
Erysipelotrichaceae Incertae Sedis	0.01
Marvinbryantia	0.01

HFD	
Clostridium	0.13
Bacteroides	0.09
Peptostreptococcaceae Incertae Sedis	0.08
Escherichia-Shigella	0.08
Parasutterella	0.05
Parabacteroides	0.03
Lachnospiraceae Incertae Sedis	0.02
Bifidobacterium	0.01
Ruminococcaceae Incertae Sedis	0.01
Anaerotruncus	0.01
Allobaculum	0.01
Erysipelotrichaceae Incertae Sedis	0.01
Family XIII Incertae Sedis	0.01

LFD+BB	
Bacteroides	0.24
Lachnospiraceae Incertae Sedis	0.10
Akkermansia	0.04
Roseburia	0.04
Parabacteroides	0.03
Ruminococcaceae Incertae Sedis	0.02
Marvinbryantia	0.02
Blautia	0.02
Subdoligranulum	0.01
Clostridium	0.01
Parasutterella	0.01
Erysipelotrichaceae Incertae Sedis	0.01
Unknown Bacteroidaceae genus	0.01

HFD+BB	
Lachnospiraceae Incertae Sedis	0.08
Bacteroides	0.07
Blautia	0.04
Clostridium	0.04
Parasutterella	0.04
Roseburia	0.04
Ruminococcaceae Incertae Sedis	0.03
Parabacteroides	0.03
Bifidobacterium	0.02
Allobaculum	0.02
Ruminococcus	0.02
Lactobacillus	0.01
Marvinbryantia	0.01
Akkermansia	0.01

Figure S2: Top ranking genera in cecum microbiota in response to each diet
